# Ureteral reconstruction using a tapered non-vascularized bladder graft: an experimental study in a canine animal model

**DOI:** 10.1186/s12894-017-0287-2

**Published:** 2017-10-23

**Authors:** Lujia Zou, Shanhua Mao, Shenghua Liu, Limin Zhang, Tian Yang, Yun Hu, Qiang Ding, Haowen Jiang

**Affiliations:** Department of Urology, Huashan Hospital, Fudan University, No.12 Wulumuqi Middle Road, Shanghai, 200040 People’s Republic of China

**Keywords:** Animal model, Bladder graft, Non-vascularized, Reconstruction, Ureter

## Abstract

**Background:**

Reconstruction of ureteral defects and strictures remains problematic for urologists. We aimed to investigate the possibility of a tapered non-vascularized bladder graft as a novel substitute for ureteral reconstruction.

**Methods:**

This experimental study was conducted on nine beagles. Under general anesthesia, a full-thickness graft with 5–6 cm in length was disassociated from the anterior upper wall of the bladder, and tapered into 1/3 to 1/2 thickness, remaining the urothelial surface. After removal of 5 cm of right-sided mid-ureter, the tapered bladder graft was tubularized along the long axis and then respectively anastomosed to the upper and lower stumps of the ureter. A retrograde urography through a cystostomy was performed 8 weeks after the ureteral reconstruction. The animals were euthanized, and histopathologic examinations of the neoureters were performed.

**Results:**

There were no severe complications during postoperative follow-up. The urography indicated patent urine excretion and no fistula or stenosis. Histopathologic examinations of the neoureters showed open lumen with urothelial lining. Nutrient vessels were observed in healthy submucosa, lamina muscularis and peripheral connective tissue.

**Conclusions:**

Our study implied that ureteral reconstruction by a tapered non-vascularized bladder graft was anatomically possible in our animal model. Further studies are expected to confirm long-term and functional outcomes.

## Background

Ureteral injuries usually arise from traumatic or iatrogenic causes [1]. In most cases, an end-to-end anastomosis without additional material can be applied for management of a short-segment ureteral defect or stricture. Furthermore, several classical surgical reconstructive methods have been well recognized, including ureteroplasty using a bladder muscle flap (Boari flap), ileal ureteral substitution, and renal autotransplantation [2].

However, these traditional methods have limitations in practical application. The Boari flap can reconstruct a limited length of distal or mid-ureter [3]. The ileal substitution has a variety of postoperative complications due to the characteristics of intestinal mucosa, including reabsorption of ammonium [4–6] and mucus secretion that may subsequently induce urinary tract infection and lithogenesis [5, 6]. Renal autotransplantation calls for a complicated procedure and is associated with possible infection and loss of renal function [1]. The Monti and Mitrofanoff techniques have been successfully developed to decrease postoperative intestinal complications, while intestinal involvement may still be related to leakage and ileus [7–9]. Concerning reconstructive materials, a bladder flap (or graft) with urothelial lining, which is similar to the ureter, seems ideal for ureteral replacement. We hypothesized that it may be possible to use a non-vascularized bladder graft for reconstruction of upper or mid-ureter. The primary concern was whether the bladder graft was able to survive without a vessel pedicle.

The main objective of our study was to investigate the possibility of a novel method using a tapered non-vascularized bladder graft to reconstruct the ureter in a canine animal model.

## Methods

This experimental study was conducted on 9 healthy male beagles (approximately 1-year-old), which were obtained from School of Agriculture, Shanghai Jiao Tong University. The mean weight of the beagles at operation time was 10.6 kg (range 8.4–12.2 kg). The dogs were housed individually in the stainless-steel cages in a controlled environment. Filtered water and a standard animal diet were available ad libitum. The study was carried out at the laboratory animal unit in School of Pharmacy, Fudan University during March to August, 2015.

The right ureter was chosen for the experimental procedures, while the left side was reserved as control. The surgical field was prepared and sterilized with povidone-iodine. Then a prophylactic dose of cefazolin was applied intramuscularly. The procedure was performed under general endotracheal anesthesia. The anesthesia was induced through intravenous administration of propofol (4 mg/kg), lidocaine (1 mg/kg), and diazepam (0.3 mg/kg) and maintained with isoflurane in 100% oxygen. The laparotomy was conducted through a full-length midline abdominal incision. Abdominal viscera were inspected to exclude any possible abnormalities, particularly of the urinary system.

A rectangular region was marked by four loose ligations at each vertex on the anterior upper wall of the filling bladder, the volume of which was approximately 30-40 mL. The region was designed with 5–6 cm in length and 1 cm in width. After full thickness resection, the bladder graft was temporarily preserved in normal saline and gently squeezed until it turned pale.

**Fig. 1:**
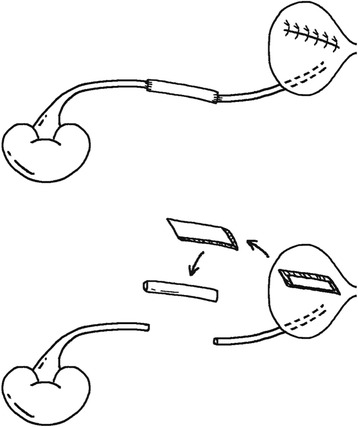
The schematic diagram of the procedure

**Fig. 2:**
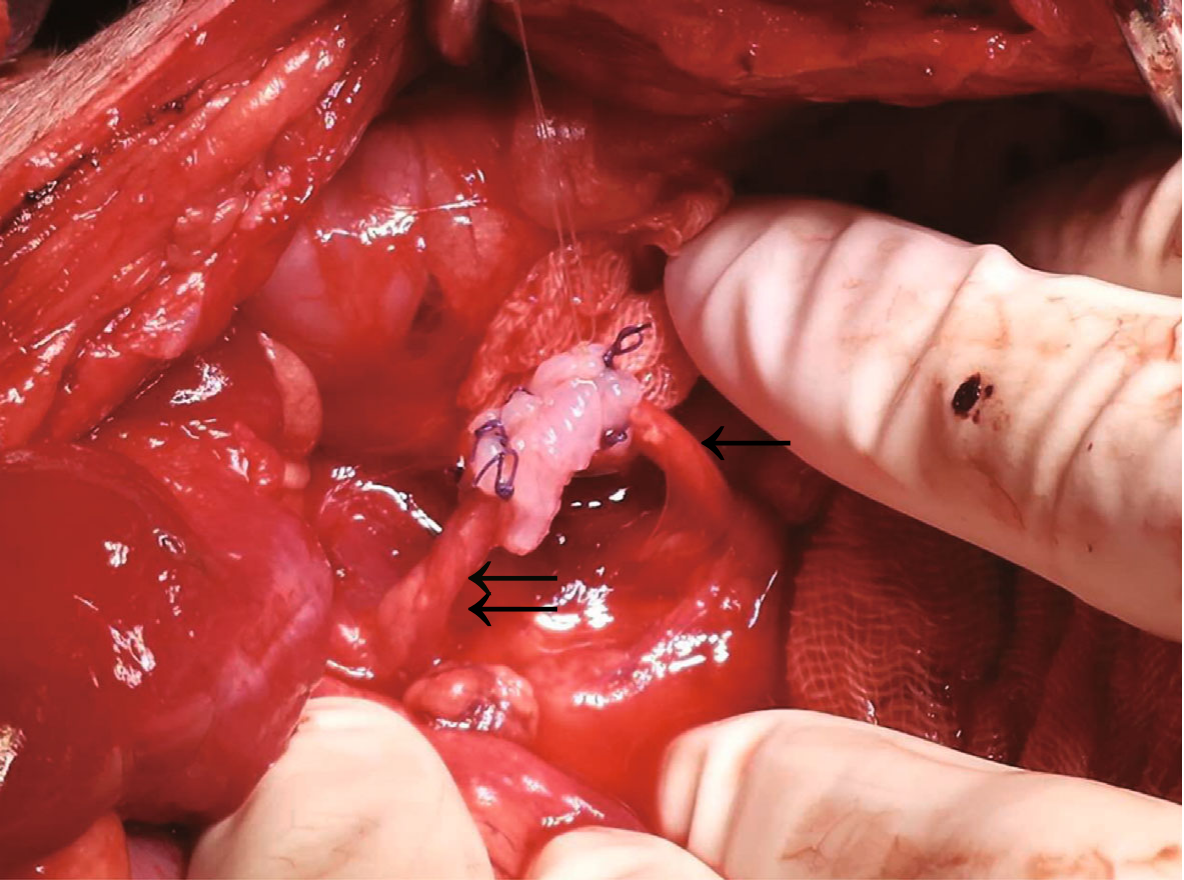
A picture of the operation. Single arrow: upper ureter. Double arrow: lower ureter

Through the cystostomy, a 4.7-Fr ureteral stent was retrogradely inserted into the right ureter from the ureteral orifice. The right ureter was mobilized from the retroperitoneal tissue, and then approximately 5 cm was removed from the middle part. The non-vascularized bladder graft was tapered carefully by an ophthalmic scissor into 1/3 to 1/2 thickness (2–3 mm), keeping the urothelial surface intact. Thereafter, the tapered bladder graft was tubularized along the long axis, with the stent inside. The two ends of the graft tube were respectively anastomosed to the upper and lower stumps of the ureter by four sutures. (Figs. 1 and 2) After the anastomoses were checked in case of hemorrhage and urine leakage, the abdominal fascia was closed by 3–4 sutures. The surgical wound was then closed layer by layer.

All animals received cefazolin and flurbiprofen for the next 3 days and cefazolin for an additional 4 days. Specific signs were monitored twice a day, including temperature, respiratory rate, pulse, appetite, activity, defecation and urination. The surgical wound was observed and sterilized at the time of monitoring. Liquid diet was started a few hours after the procedure, and it was advanced to regular diet the next day. The ureteral stent was maintained for the postoperative 6 weeks and removed through a brief cystostomy. 8 weeks after the reconstructive procedure, a retrograde urography by a 5-Fr tube was performed through another cystostomy to assess the possibility of leakage or stenosis.

The animals were then euthanized by intravenous injection of potassium chloride under general anesthesia. After thorough exploration of the abdominal cavity and surgical field, the right-sided kidney, ureter (together with peripheral connective tissue) and part of the bladder were resected and fixed with formalin immediately. The specimens were embedded with paraffin and stained with hematoxylin & eosin. Two experienced genitourinary pathologists separately finished the microscopic examination.

## Results

At the end point of the experiment, all the 9 dogs tolerated the procedure and survived. All had regular eating and activities. A subcutaneous abscess at the abdominal incision occurred to one dog, and was solved by a surgical debridement. There were otherwise no obvious signs of complications including severe gross hematuria, peritoneal infection or urine leakage. The ureteral stents in 7 animals were in place till the removal, and had been delivered out of the urinary tract in the other two. No obvious alteration of micturition rhythm was observed.

Through the abdominal exploration after the euthanasia, there was no hematoma, infection or urine leakage in the abdominal cavity. The reconstructive segments of the right ureters were intensely wrapped by fibrous adhesion. Mild hydronephrosis was observed in the right kidneys and upper ureters. Through the second cystostomy, urine was observed continuously outflowing from the orifices of the right ureters.

**Fig. 3:**
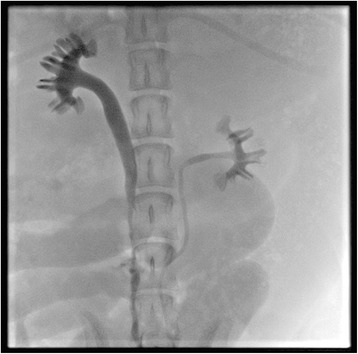
The urography showing patent urine excretion and no fistula or stenosis (the left side as control)

During the retrograde urography, the lumen of the neo-ureters easily accepted the 5-Fr tubes. The urography indicated normal ureteral caliber and patent urine excretion, without obvious fistula or stenosis. Grade of hydronephrosis assessed through the urography was accordant with the gross examination (Fig. 3).

**Fig. 4:**
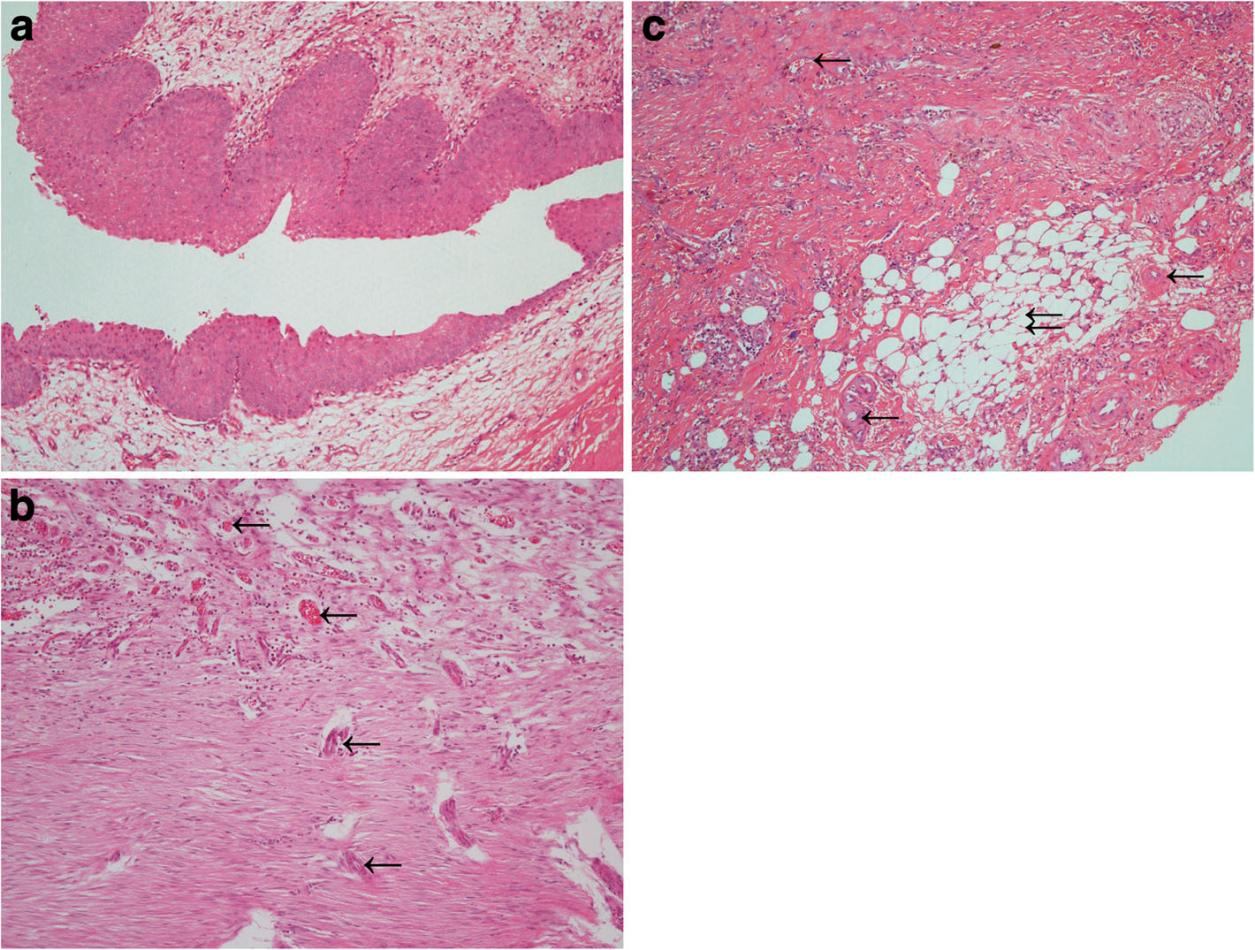
Histological examination of reconstructive ureter (H.E.). **a** Longitudinal section view showing open lumen with complete urothelial lining in the neo-ureter. (×10) **b** The healthy submucosa and lamina muscularis with nutrient vessels. (×10) Single arrow: nutrient vessels in submucosa and lamina muscularis. **c** The disordered smooth muscle fibers, without a serosa between the lamina muscularis and connective tissue; nutrient vessels in the periureteral connective tissue. (×10) Single arrow: The nutrient vessels of the reconstructive ureter. Double arrow: the periureteral connective tissue

The microscopic examination of the reconstructive ureteral segments showed that the open lumen was almost completely covered with pseudostratified urothelial lining. The urothelial cells formed the arrangement of 5–7 rows, similar to that of an empty bladder. In the denuding area, the basement membrane of urothelium was also complete. The submucosa and lamina muscularis were alive and healthy with abundant nutrient vessels. Meantime, infiltration of inflammatory cells was observed in the submucosa. The smooth muscle fibers had disordered trends near the periureteral connective tissue, without serosa between the two layers. Nutrient vessels were also noticed in the periureteral connective tissue (Fig. 4).

## Discussion

Ureteral injuries usually require surgical management, except for ureteral contusion and perforation. A short segment of ureteral defect can be repaired by end-to-end anastomosis, while a long-segment defect usually calls for advanced procedures and additional materials to reconstruct the continuity of injured ureter [1, 10]. Tissue lined with urothelium may be the ideal material for ureteral reconstruction [1]. The reasons are associated with the characteristics of urothelial lining, including no reabsorption of ingredients of urine and no mucus secretion that may cause infection and lithogenesis. In brief, the urothelial lining can maintain the normal physiological functions of the ureter. The technique of a Boari flap is a classical example [3].

By contrast, intestinal or ileal substitution was regarded as the most accepted procedure for long-segment ureteral reconstruction so far. The advantages include adequate substitutive material and available blood supply for long-segment reconstruction [11]. However, it also involves numerous postoperative complications [12]. The absorptive and secretory functions of intestinal mucosa may lead to urine leakage, infection, anastomotic stenosis, lithogenesis, electrolyte and/or acid-base imbalance, and even renal function loss. The oversized caliber may exacerbate hydronephrosis and consequently accelerate impairment of renal function [4–6, 12]. Interference to the gastrointestinal tract can also bring about postoperative problems [12]. The development of the Monti and Mitrofanoff techniques is an important improvement of intestinal substitution. Some previous studies claimed that the techniques were effective for ureteral replacement with sustained promising long-term results and consequent absence of metabolic complications [7, 8]. On the other hand, some studies reported the postoperative intestinal complications such as ileus and leakage, which called for second operations sometimes [8, 13].

Novel methods using different materials for ureteral reconstruction have been developed, mostly avoiding involvement of intestinal mucosa. An intestinal seromuscular tunnel [1] and a seromuscular tapered ileal tube [10] were reported to form neo-ureter with complete urothelial lining of the inner surface. Previous studies demonstrated that it was feasible to rebuild normal urothelial lining of the reconstructive segment [14, 15]. Moreover, an intestinal seromuscular segment with autograft of bladder mucosa was also proved applicable for ureteral reconstruction [16]. However, demucosalized intestinal segments may be complicated by shrinkage and regrowth of original gastrointestinal epithelium [17]. The efficacy of modified methods using intestinal segments demand further investigation. An autologous graft of granulation tissue capsule was also mentioned as a new material for ureteral substitution [12].

Recently, a spiral bladder muscle flap with vascular pedicles was designed to reconstruct full-length ureteral defects in 6 patients [2]. This procedure can avoid complex preoperative preparation and metabolic complications caused by intestinal substitution. It was claimed that the procedure can retain peristaltic function of bladder smooth muscle and consequently prevent dilation, fluid accumulation and lithogenensis. However, the authors also noted that kidney descent and fixation and a psoas hitch should be performed simultaneously to improve the success rate. Furthermore, preservation of superior vesical arteries required extremely subtle surgical techniques, which may lead to increased postoperative complications and limited popularization.

Considering its good compliance for the experimental surgery and resistance to infection, we selected beagles to establish the animal model. In our study, the gross examination and the urography identified that the neo-ureters were patent, only with mild hydronephrosis, which probably resulted from urine reflux due to the ureteral stent. The microscopic examination proved that the tapered bladder grafts were survivable without a vessel pedicle. Partial loss of urothelial lining, which was quite slight and local, may result from the retrograde catheterization and instillation of the contrast agent. The intact basement membrane allowed the healing of urothelium and thus avoided ureteral stenosis. Based on our histologic findings, we supposed that the nutrient vessels from periureteral connective tissue were sufficient to nourish the graft. Theoretically, the tapering procedure can reduce the volume of the graft tissue, and consequently decrease the nutrient demand. Meanwhile, it can create a raw surface of smooth muscle, cause inflammatory exudation and thus increase nutrient vessels and attachment between the graft and peripheral connective tissue. Our preliminary experience implied that a tapered non-vascularized bladder graft was a novel possible substitute material for ureteral reconstruction.

Another concern arose from the different capacity of bladder between the animal model and human beings. According to the previous studies, a 10-kg-heavy dog has a bladder capacity of approximately 40–70 ml [18, 19], while an adult human being has 300–500 ml. Comparatively, the beagles and human beings have a similar bladder capacity with respect to their own weight. Alteration of micturition rhythm was not observed in the animals, which indicated that the bladder capacity was not apparently influenced by the procedure. There was no concern about the application of our procedure for ureteral reconstruction in human beings.

The potential clinical application of this procedure is reconstruction of defect and stricture in upper and mid-ureter, for which a psoas hitch or a Boari flap is unsuitable. The bladder has sufficient substituted material for required length of the neo-ureter. However, the primary limitations of our study were a small sample size and a short-term follow-up. Therefore, the efficacy and safety of this procedure must be confirmed by a larger sample size and longer postoperative follow-up assessments.

## Conclusion

Reconstruction of ureteral defect and stricture maintains a troublesome problem for urologists. Additional to the traditional intestinal substitution, a number of new procedures and materials have been developed for ureteral reconstruction. Our findings showed that it was anatomically possible to use a tapered non-vascularized bladder graft for ureteral reconstruction in the experimental animal model. In consideration of the limitations of our study, further investigations with a larger sample size are expected to evaluate the long-term functional efficacy and safety.
